# The Elevated Susceptibility to Diabetes in India: An Evolutionary Perspective

**DOI:** 10.3389/fpubh.2016.00145

**Published:** 2016-07-07

**Authors:** Jonathan C. K. Wells, Emma Pomeroy, Subhash R. Walimbe, Barry M. Popkin, Chittaranjan S. Yajnik

**Affiliations:** ^1^Childhood Nutrition Research Centre, UCL Institute of Child Health, London, UK; ^2^McDonald Institute for Archaeological Research, University of Cambridge, Cambridge, UK; ^3^Maharashtra Association of Anthropological Sciences, Pune, India; ^4^Nutrition Department, Gillings Global School of Public Health, University of North Carolina School of Public Health, Chapel Hill, NC, USA; ^5^Diabetes Unit, King Edward Memorial Hospital and Research Centre, Pune, India

**Keywords:** India, South Asia, thrifty phenotype, diabetes, evolution

## Abstract

India has rapidly become a “diabetes capital” of the world, despite maintaining high rates of under-nutrition. Indians develop diabetes at younger age and at lower body weights than other populations. Here, we interpret these characteristics in terms of a “capacity–load” model of glucose homeostasis. Specifically, we assume that glycemic control depends on whether the body’s “metabolic capacity,” referring to traits, such as pancreatic insulin production and muscle glucose clearance, is able to resolve the “metabolic load” generated by high levels of body fat, high dietary glycemic load, and sedentary behavior. We employ data from modern cohorts to support the model and the interpretation that elevated diabetic risk among Indian populations results from the high metabolic load imposed by westernized lifestyles acting on a baseline of low metabolic capacity. We attribute this low metabolic capacity to the low birth weight characteristic of Indian populations, which is associated with short stature and low lean mass in adult life. Using stature as a marker of metabolic capacity, we review archeological and historical evidence to highlight long-term declines in Indian stature associated with adaptation to several ecological stresses. Underlying causes may include increasing population density following the emergence of agriculture, the spread of vegetarian diets, regular famines induced by monsoon failure, and the undermining of agricultural security during the colonial period. The reduced growth and thin physique that characterize Indian populations elevate susceptibility to truncal obesity, and increase the metabolic penalties arising from sedentary behavior and high glycemic diets. Improving metabolic capacity may require multiple generations; in the meantime, efforts to reduce the metabolic load will help ameliorate the situation.

## Introduction

Although chronic diseases were initially labeled “diseases of affluence,” due to their association with urban living and westernized behavior, they have rapidly become an epidemic in many middle-income countries that have yet to resolve chronic under-nutrition (1–3). India, where over 40% of children under 5 years are malnourished (4), has also become known as a “diabetes capital” of the world, with an estimated 65+ million diabetic patients aged 20–79 years in 2013, and substantial further increases anticipated (5). Other south Asian countries show a similar epidemiology (6). Urbanization, with its many effects on behavior, is a key driver of the Indian diabetes epidemic (7–9), but numerous studies further indicate that south Asians have elevated diabetes susceptibility compared to other populations (10–12). Emigrant populations of Indians in high-income countries have higher diabetes rates than other ethnic groups (13), and this difference remains if adjustment is made for classic risk factors (14).

There is little consensus on the causes of the Indian diabetes epidemic, or on the appropriate public health policies by which the issue may be addressed. The simplest perspective might invoke genetic susceptibility. The influential “thrifty genotype” hypothesis proposed that population metabolic differences have arisen through differential ancestral exposure to cycles of “feast and famine” (15), generating differential susceptibility to diabetes in modern environments. The molecular basis of type 2 diabetes is polygenic: over 100 genes have already been associated with the condition, with the magnitude of effect of each gene typically very small (16). Studies are increasingly testing the hypothesis that population-specific risk-alleles contribute to the elevated prevalence of diabetes in South Asians, but current evidence is limited, and the findings vary according to the investigative approach used.

Most risk-alleles appear to exert similar directions and magnitudes effects in European and South Asian populations (17, 18), but it is important to consider whether this applies universally, and whether the frequency of such risk-alleles varies between populations. One global study reported decreasing frequencies of type II diabetes risk-alleles with increasing geographical distance from Africa toward East Asia, suggesting a diabetes-protective consequence of migration (19). This would predict a lower genetic risk in South Asians relative to Europeans, which is contrary to the empirical scenario of their higher diabetes prevalence. Conversely, another study identified diabetes risk-alleles in South Asian populations that were not associated with diabetes risk in European populations (20). More generally, genes related to energy/lipid metabolism represent one of three key groups of genes that show unique South Asian alleles or allelic stratification compared with Europeans, along with those associated with immune function and skin/hair pigmentation (21). Since such genotypes relating to both immune function and skin/hair phenotype can be linked to specific selective pressures affecting South Asian populations, i.e., diseases endemic to the Indian subcontinent and ultraviolet radiation, respectively (21), it is logical that the metabolism-related variants are similarly a response to long-term selective pressures.

There is also some evidence that important shared risk-alleles may act differently among South Asians compared with other populations. The *FTO* (fat mass- and obesity-associated) gene is associated with obesity and diabetes risk in Europeans, Africans, and Asians (22–26), but the minor (obesity-associated) FTO allele is both less common (30–33 vs. 42%) and explains less variation in body mass index (BMI) (0.20 vs. 0.34%) among South Asians compared with Europeans (27, 28). It may be that the relationship is weaker in the former as BMI represents a different quantity and distribution of body fat (25, 29). Furthermore, the effect of FTO appears to be environment dependent among South Asians, resulting in a much stronger relationship with obesity and diabetes among urban relative to rural populations (22, 25).

Collectively, these data indicate that the heightened diabetes risk of South Asians is very likely to include a genetic component, but it is likely to be relatively small. Going beyond the genes of humans themselves, there is accumulating evidence for the role of the gut microbiome in influencing diabetes and obesity risk (30), and a recent meta-analysis highlighted distinctions between the gut microbiota of Indians and Bangladeshis compared with North Americans, which may have implications for chronic disease risk (31). Further work may shed more light on how the genes of other species impact human metabolism in population-specific ways.

**Figure 1:**
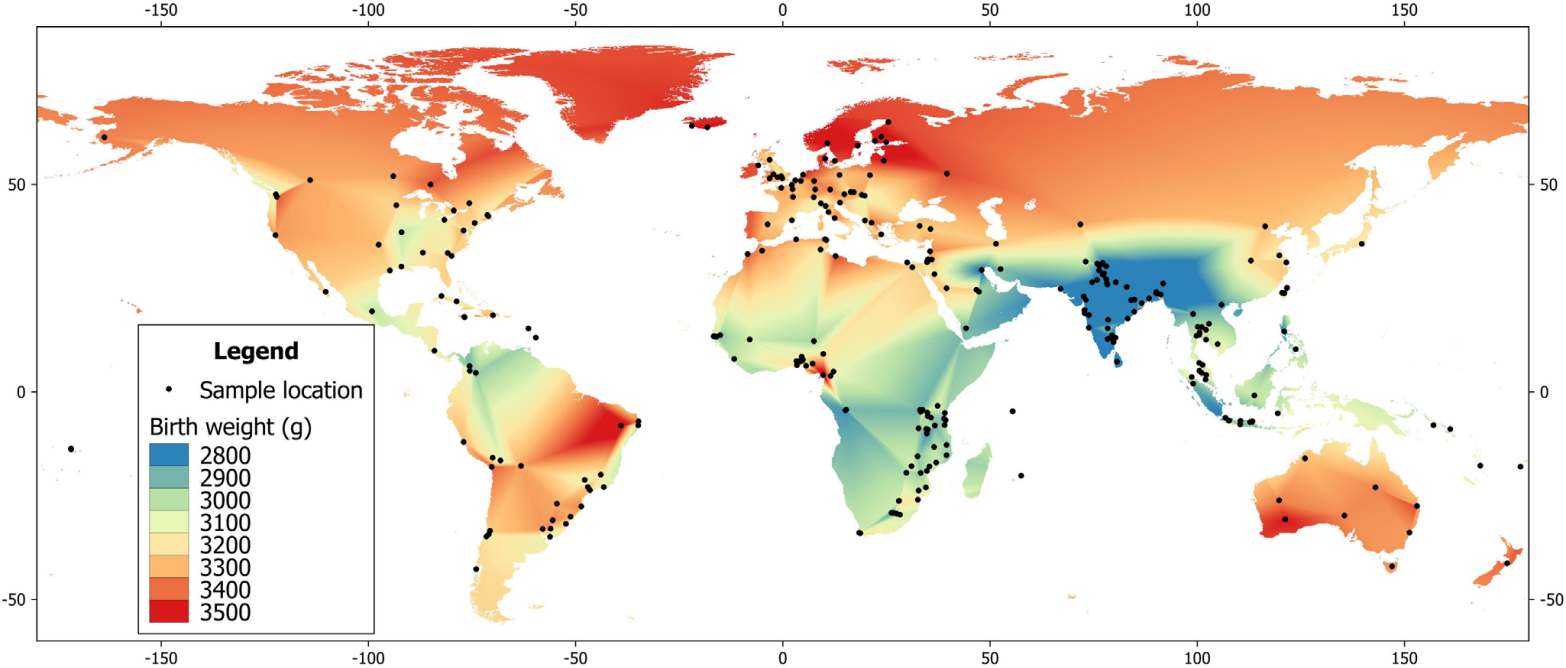
**Global heat map of mean birth weight, based on data from the World Health Organization (35)**.

More generally, the primary underlying mechanism is likely to comprise epigenetic effects, many of which originate in early life (the Developmental Origins of Adult Disease hypothesis), though some may continue to emerge in adolescence and later life. Epigenetic influences are demonstrated for pre-conceptual and gestational nutritional and metabolic factors. For example, the influential “thrifty phenotype” hypothesis emphasizes a link between life-course plasticity and diabetes risk, proposing that poor growth in early life reduces glucose tolerance in adulthood (32). Low birth weight and weight at 1 year have been associated in a number of studies with subsequent diabetes risk, though the risk may also increase at higher birth weight (33, 34). Consistent with the hypothesis that early growth retardation increases diabetes risk, birth weight in India is among the lowest globally (Figure 1) (35), and variability in maternal phenotype and birth weight have been associated with elevated diabetes risk in Indian birth cohorts (36, 37).

Nonetheless, it remains unknown why low birth weight and elevated diabetic risk should persist among populations of South Asian ancestry despite improvements in living standards, and across multiple generations after migration to more affluent environments. Why, for example, do South Asian populations have a higher ratio of fat to lean mass than most other populations, as described below? Understanding the origins of the Indian phenotype may have implications for designing effective strategies to reduce chronic disease risk among people of South Asian ancestry worldwide. A similar scenario may apply to many other ethnic groups from low- and middle-income countries, which typically have elevated rates of diabetes relative to Europeans living in high-income settings (38).

The aim of this paper is to develop an evolutionary perspective on the elevated susceptibility to diabetes in South Asian populations. First, we describe a metabolic model of diabetes risk, characterized by the balance between the “metabolic capacity” to maintain glycemic control, and the “metabolic load” generated by factors such as obesity, unhealthy diet, and sedentary behavior. Diabetes occurs when the metabolic load exceeds the capacity to maintain fuel homeostasis. We show that height and physique can act as reliable markers of metabolic capacity, and illustrate how this applies to the contemporary South Asian phenotype. Then, using archeological and historical evidence, we argue that long- and short-term chronic energy stress and low dietary protein availability have induced major downward trends in metabolic capacity, indexed by decreasing stature and lean mass. These traits, and their life-course emergence, are strongly associated with the elevated diabetes risk experienced among contemporary Indians exposed to the rapid lifestyle changes accompanying urbanization. In other words, the elevated susceptibility to diabetes in South Asian populations can be attributed to rapid increases in metabolic load *exposing* the long-term decline in metabolic capacity.

## A Metabolic Model of Diabetes

Type 2 diabetes is a condition in which failure to regulate blood glucose levels by the hormone insulin leads to tissue damage, elevated cardio-metabolic risk, and, in the absence of treatment, increased risk of premature death. By the mid-twentieth century, clinical research in industrialized countries had led to consensus that diabetes was an “adult-onset” disease, primarily attributable to genetic profile and adult lifestyle. The strongest risk factor was obesity, considered the dominant cause in around 80% of cases (39). Subsequent research led to the concept of diabetes as a “two-hit” disease, involving both insulin resistance in muscle tissue, and failure of the pancreatic beta-cells to produce enough insulin to compensate for this resistance (40).

The consensus view of diabetes as an adult-onset disease underwent a paradigm shift in the 1990s, when the “thrifty phenotype” hypothesis was published (32). These authors proposed that fetal and infant under-nutrition reduced growth of the pancreas and muscle tissue to protect the growing brain, at the cost of reduced glucose tolerance in adulthood (32). They therefore highlighted the additional contribution of development to variability in diabetes risk.

Data increasingly support the thrifty phenotype hypothesis: in both European and Asian populations, famine exposure *in utero* (41, 42) and low birth weight (34, 36) are associated with adult glucose intolerance, while the adverse consequences of low birth weight are enhanced in the presence of high BMI or adult adiposity (43–45). Low birth weight and malnutrition during early post-natal life are associated with beta-cell dysfunction (46–49), which impairs glucose tolerance. This susceptibility to glucose intolerance is exacerbated by subsequent catch-up growth in early childhood, which elevates adiposity and promotes insulin resistance (50–52). The environmental etiology of type 2 diabetes appears strongly mediated by oxidative stress, which, on the one hand, provokes insulin resistance (53, 54), and, on the other hand, contributes to beta-cell damage and eventual deficiency in insulin secretion (55, 56).

While the thrifty phenotype hypothesis initially focused on the consequences of low birth weight, however, studies in Europeans show inverse dose–response associations of birth weight with later glucose tolerance across the range of birth weight, with similar associations evident for thinness (low ponderal index) and length at birth (34, 43, 57), although diabetes risk increases again at high birth weights in some populations (33).

Recognizing these broad dose–response relationships, we have built on the thrifty phenotype hypothesis by proposing a continuous model of disease risk, emphasizing the interaction of two fundamental traits: metabolic capacity, referring to phenotypic traits indexing the capacity for homeostasis, and metabolic load, referring to phenotypic traits that challenge homeostasis (58). For diabetes, the most relevant components of metabolic capacity are the function of the pancreas (responsible for producing insulin) and muscle mass (influencing glucose clearance rate), each of which is strongly contingent on fetal and infant growth (32, 59). The most relevant components of metabolic load are adiposity, dietary glycemic load, and sedentary lifestyle, all of which perturb normal glycemic control and promote chronic inflammation, deleterious to beta-cell function (60, 61).

**Figure 2:**
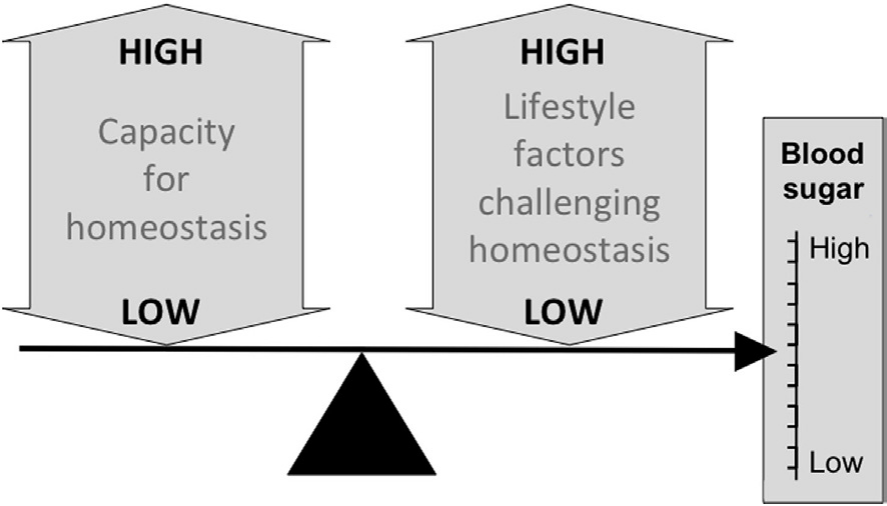
**Schematic diagram illustrating the basic capacity–load model of glycemic control, in which blood sugar levels rise in association with traits such as a high glycemic diet, sedentary behavior and, high levels of body fat, and decrease in proportion to the homeostatic capacity of the body, indexed by traits such as pancreatic beta-cell mass and muscle mass**.

**Figure 3:**
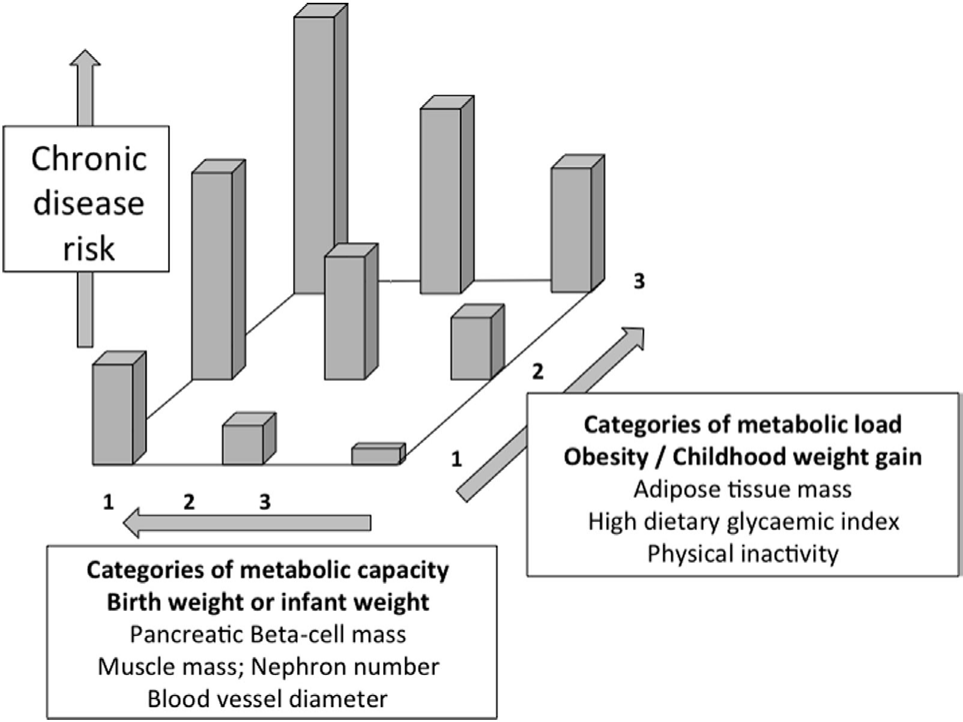
**Schematic diagram illustrating the capacity–load model of metabolic dysfunction**. Both decreasing metabolic capacity and increasing metabolic load independently increase the risk of glucose intolerance and overt diabetes. The highest risk is, therefore, found in those with high load and low capacity. Redrawn with permission from Ref. (58).

**Figure 4:**
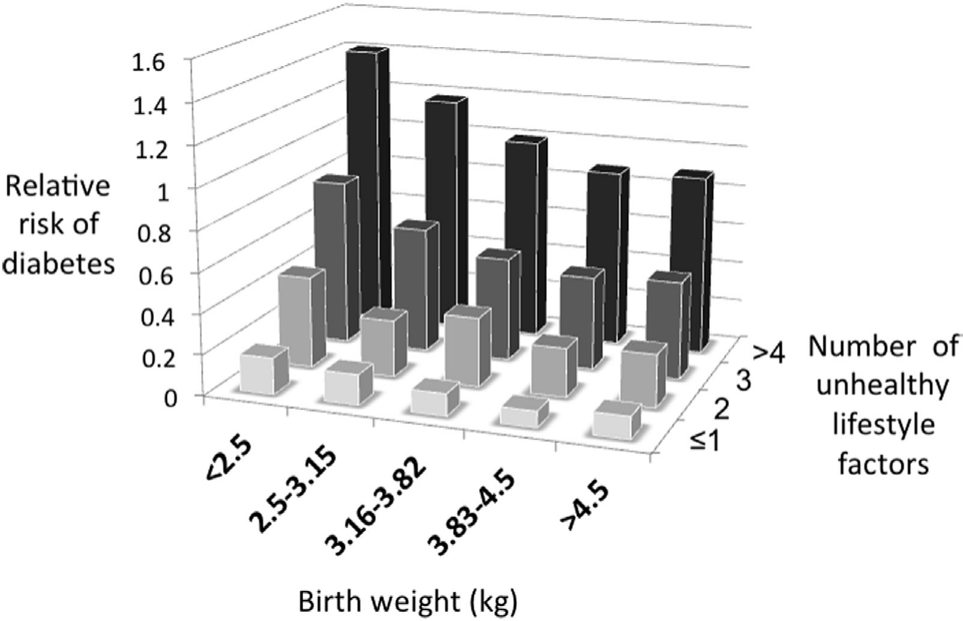
**The capacity–load model illustrated for the prospective risk of developing diabetes in three US cohorts**. Data taken from Ref. (45).

Figure 2 illustrates the basic model, showing how the relationship between capacity and load on an immediate basis impacts the regulation of blood sugar levels. Variability in the relationship between capacity and load over the longer-term then shapes the risk of developing diabetes (Figure 3). Figure 4 provides supporting evidence for this model from three US cohorts. Consistent with the model, birth weight is inversely associated with diabetes risk across its entire range, while “components of an unhealthy adult lifestyle,” including high BMI, are positively associated with diabetes risk. The highest risk of diabetes is, therefore, found in those born small who lead a particularly unhealthy adult lifestyle, promoting the development of abdominal obesity. Importantly, these data support a key prediction of the model, namely that the diabetic risk elicited by low birth weight is relatively low in the absence of metabolic load, but greatly exacerbated in the presence of a high metabolic load.

Information on metabolic capacity and load could, therefore, help assess diabetes risk on a routine basis. However, especially in low- and middle-income countries, data on birth weight are likely to be lacking in the vast majority of adult individuals. A solution to this dilemma is to obtain data on traits that are strongly correlated with birth weight. Numerous studies have demonstrated positive associations between birth weight and rankings of height and lean mass throughout the life course (59, 62–64), including in Indian populations (65). Weight and height during infancy are also predictive of adult stature and lean mass (59) This means that adult height can act as a valuable proxy for growth during fetal life and infancy and, hence, for metabolic capacity. Likewise, although accurate assessment of adult body composition is not feasible on a routine basis, BMI or waist circumference adjusted for height can act as simple markers of metabolic load, as can behavioral markers, such as physical inactivity.

**Figure 5:**
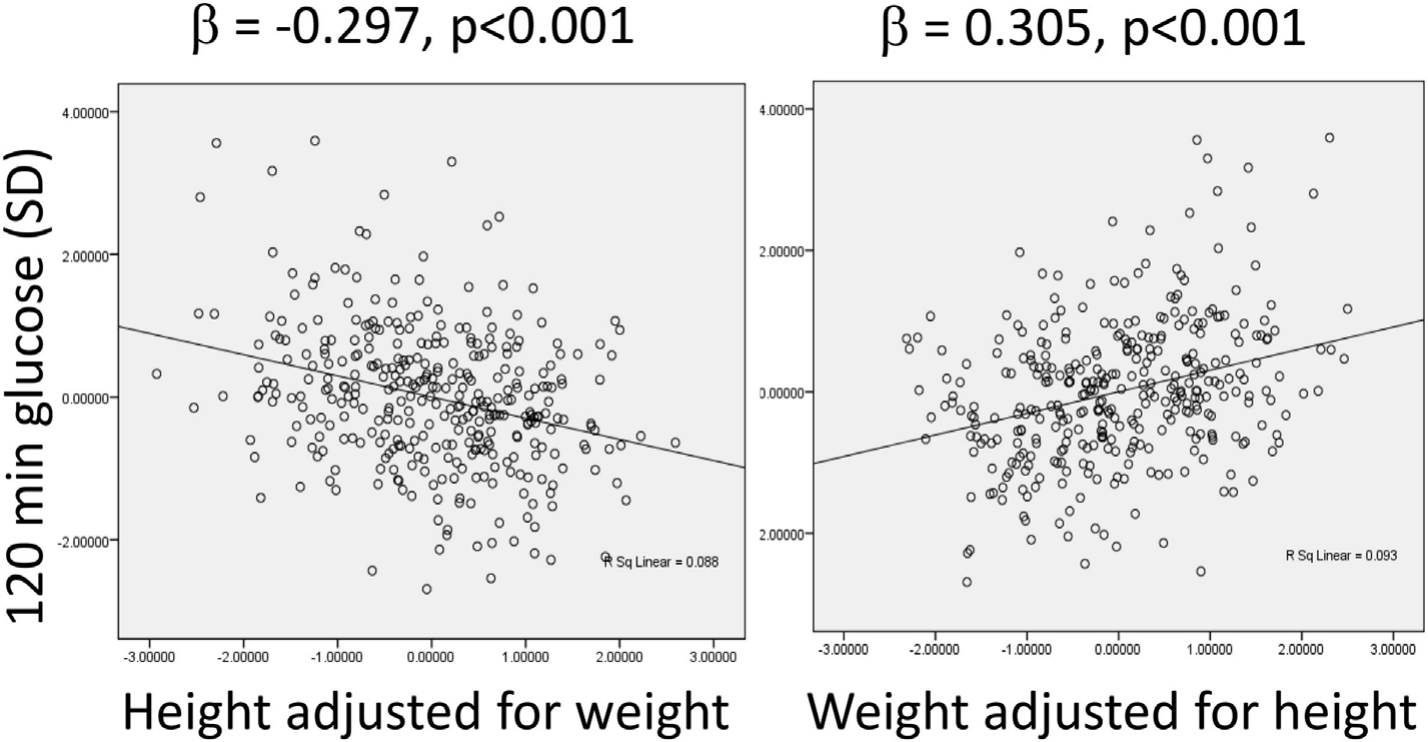
**Associations between plasma glucose levels 120 min after administration of an oral glucose load, a marker of glucose intolerance, with height adjusted for weight, and weight adjusted for height in an urban Indian population at the age of 21 years**. Data taken from Ref. (67).

Support for this approach is shown in Figure 5, which presents data from an urban population at 21 years of age from Pune, west central India. In this cohort, adjusting for weight, stature shows a dose–response negative association with plasma glucose at 120 min following administration of an oral glucose load. This indicates that stature “promotes” glucose tolerance, and acts as a simple marker for metabolic capacity, most likely because both stature and beta-cell mass are sensitive to under-nutrition in fetal life and infancy (59, 66). Conversely, independent of stature, glucose tolerance is negatively associated with body weight, a simple index of metabolic load. Thus, taller stature is diabetes-protective, while higher body weight increases risk.

Data from a number of other studies broadly support the link between stature and diabetic risk (Table 1), though there is uncertainty as to whether this elevated risk derives directly from early-life growth constraint, or its association with reduced muscle mass, resulting in poorer glucose tolerance during standard tests (68). The association appears especially strong among women, particularly during pregnancy. A recent meta-analysis of studies reporting relative risk of diabetes in relation to shorter stature that included a subset of the studies in Table 1 found a significant association among women but not men (69). Interestingly, the authors suggested that the relationship between diabetes risk and short stature was stronger among populations of Asian and Australian origin than among Africans and Europeans. Overall, this evidence justifies the use of stature as a simple marker of metabolic capacity in populations across time and geography. Below, we use this approach to reconstruct potential trends in metabolic capacity over lengthy time periods in the history of India.

**Table 1:** **Reported risk of type 2 diabetes in men, women, or sexes combined, or gestational diabetes in women, associated with tall stature**.

Reference	Country	*n*	Participant age (years)	Risk	95% CI	Comparison and test
**Men**						
Liu et al. (70)	USA	3128	20–85	0.91	0.75–1.10	Per SD increase (OR)
Lorenzo et al. (71)	USA	730	25–64	1.14	0.85–1.51	Per 1 SD increase (OR)
Schulze et al. (72)	Germany	9711	40–65	0.71	0.53–0.95	Highest vs. lowest quintile (RR)
Han et al. (73)	Netherlands	5887	20–59	0.23	0.09–0.79	Highest vs. lowest tertile (OR)
Njolstad et al. (74)	Norway	6098	35–52	1.15	0.97–1.35	Per 5 cm increase (RR)
Kumari et al. (75)	UK	5807	35–65	0.65	0.50–0.90	Highest vs. lowest tertile (OR)
Bozorgmanesh et al. (76)	Iran	1589	>20	0.95	0.67–1.35	Per 1 SD increase (HR)
Janghorbani and Amini (77)	Iran	614	30–60	0.81	0.39–1.68	Highest vs. lowest quartile (OR)
**Women**						
Liu et al. (70)	USA	3060	20–85	0.99	0.82–1.21	Per 1 SD increase (OR)
Lorenzo et al. (71)	USA	1000	25–64	0.88	0.70–1.11	Per 1 SD increase (OR)
Schulze et al. (72)	Germany	15,402	35–65	1.10	0.78–1.55	Highest vs. lowest quintile (RR)
Han et al. (73)	Netherlands	7018	20–59	0.62	0.31–1.22	Highest vs. lowest tertile (OR)
Njolstad et al. (74)	Norway	5556	35–52	0.71	0.58–0.87	Per 5 cm increase (RR)
Lawlor et al. (78)	UK	4286	60–79	0.91	0.80–1.03	Per 1 SD increase (OR)
Kumari et al. (75)	UK	2579	35–65	0.82	0.50–1.40	Highest vs. lowest tertile (OR)
Branchtein et al. (79)	Brazil	5564	27.8 ± 5.5	0.63	0.45–0.87	Highest vs. lowest quartile (OR)
Bozorgmanesh et al. (76)	Iran	2132	>20	0.62	0.46–0.83	Per 1 SD increase (HR)
Janghorbani and Amini (77)	Iran	1754	30–60	0.97	0.59–1.58	Highest vs. lowest quartile (OR)
**Combined sexes**						
Eckel et al. (80)	Germany	2733	35–64	0.95	0.89–1.00	Per 1 cm increase (HR)
Janghorbani and Amini (81)	Iran	1092	42.8 ± 6.4	0.54	0.31–0.93	Highest vs. lowest quartile (RR)
Schooling et al. (82)	China	10,304	50+	1.04	0.99–1.10	Per 1 SD increase (OR)
Veena et al. (83)	India	509	46	0.99	0.97–1.01	Per 1 cm increase (OR)
Sayeed et al. (84)	Bangladesh	6847	15+	0.57	0.37–0.86	≥ 165 vs. 130–149 cm (OR)
Hoque et al. (85)	Bangladesh	7565	35+	0.82	0.69–0.98	Highest vs. lowest quartile (OR)
**Gestational diabetes mellitus**						
Rudra et al. (86)	USA	1644	–	0.40	0.17–0.95	>170 vs. ≤160 cm (RR)
Brite et al. (68)	USA	13,5861	–	0.80	0.78–0.82	Per 5 cm increase (OR)
Ogonowski and Miazgowski (87)	Poland	2841	–	0.96	0.94–0.97	Per 1 cm increase (OR)
Jang et al. (88)	Korea	9005	–	0.49	0.33–0.73	Highest vs. lowest quartile (OR)

*OR, odds ratio; RR, relative risk; HR, hazard ratio. Values in shaded cells are significant p < 0.05*.

The capacity–load model offers a specific explanation for why reduced metabolic capacity has only led to an epidemic of diabetes and associated diseases in India in recent decades. If metabolic load remains relatively low, through the consumption of traditional diets and maintenance of high physical activity levels, then it will not outstrip metabolic capacity, and conditions such as diabetes should not manifest. This scenario is consistent with data from various populations characterized by relatively short stature, indicating low birth weight, which maintained traditional lifestyles through the twentieth century and demonstrated negligible rates of diabetes (89–91). It is the superimposition of high metabolic load associated with urbanization (sedentary behavior, diets high in fats and refined carbohydrates) on the background of low metabolic capacity that is predicted to lead to metabolic dysfunction (92).

## The Contemporary South Asian Phenotype from a Capacity–Load Perspective

**Figure 6:**
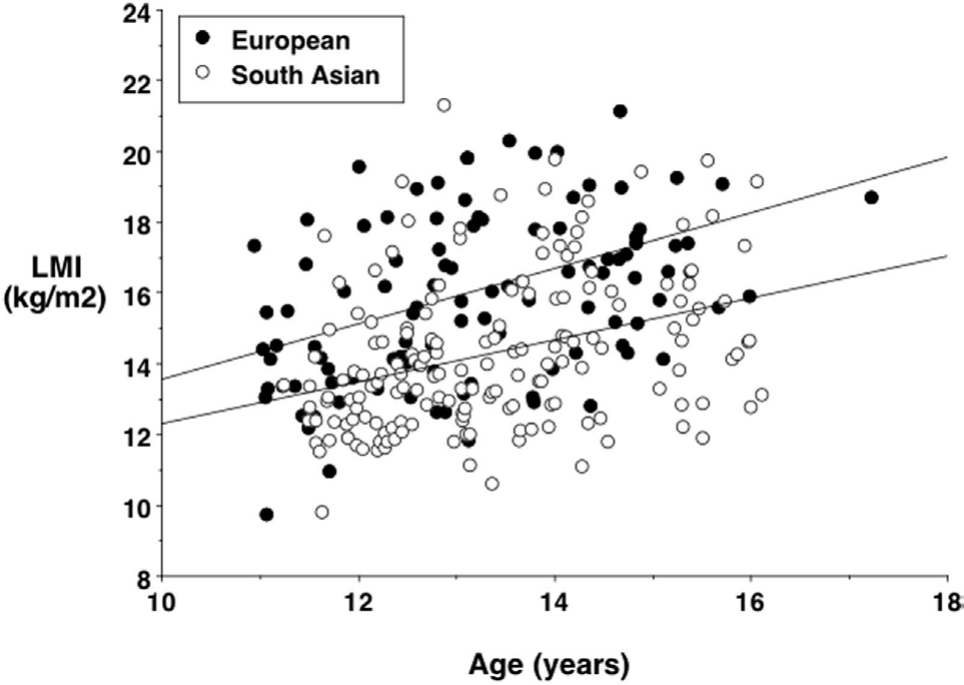
**Lean mass index (lean mass divided by height squared) against age in European and South Asian adolescents living in London, UK, showing a systematic trend for lower values in the South Asians**. Data taken from Ref. (96).

More detailed studies provide substantial support for the capacity–load model of diabetes risk in South Asian populations. Numerous studies have shown that, compared to Europeans, Indians are not only shorter but also have reduced levels of lean mass (93–95). For example, Figure 6 illustrates the association between age and lean mass adjusted for height in South Asian and European children and adolescents from London, UK. There is a substantial reduction in lean mass relative to height in the South Asian sample at all ages.

**Figure 7:**
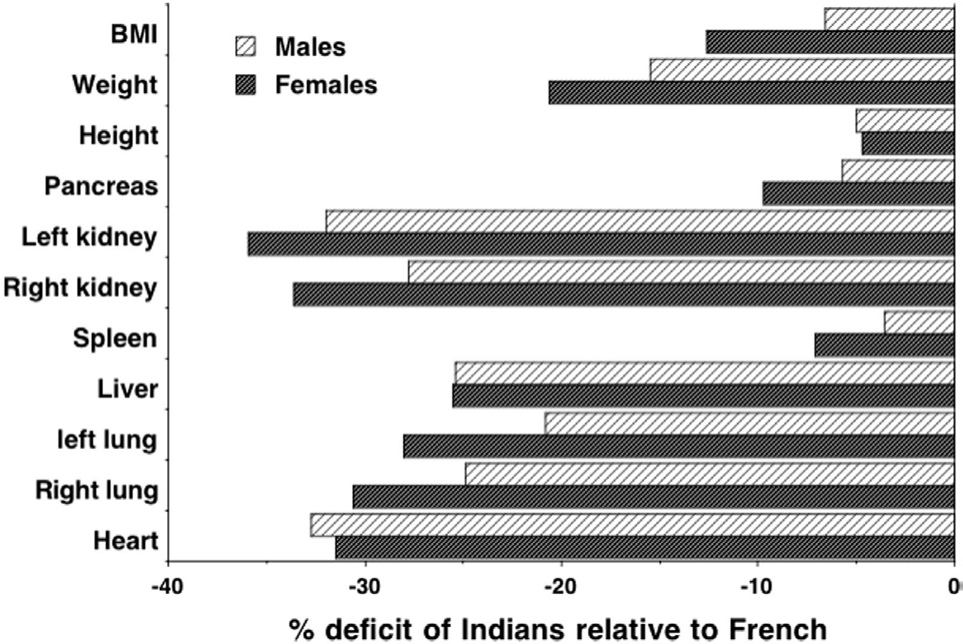
**Data on height-adjusted organ masses from autopsies of Indian and French adults, expressed as % deficit of Indians relative to French**. Data taken from Ref. (100, 101).

Such lean mass deficits may contribute causally to increased diabetes risk, for example, through decreased glucose clearance and possibly earlier beta-cell exhaustion (97). Indeed, more specific data on organ size indicates substantial reductions in South Asians relative to Europeans. Organs have a higher metabolic rate per unit mass than muscle tissue (98) and, therefore, contribute disproportionately to basal energy metabolism. Previous work has shown that organ mass scales with stature squared in humans (99). Figure 7 illustrates the percentage reductions in the size of various organs of Indians relative to Europeans, adjusting for this scaling association (100, 101). Most organs are significantly reduced in Indians, but the pancreas and spleen, associated with metabolic sensitivity and immune system, respectively, are only slightly smaller than those of Europeans. These data suggest a particular version of the “thrifty phenotype” in south Asians, which may indicate adaptation to local ecological conditions as discussed in more detail below. Intriguingly, very similar findings emerged from a study that exposed rats to chronic malnutrition over 50 generations. Compared to control rats, mass of the heart and muscle tissue was reduced in those undernourished, whereas liver and pancreas mass were not significantly lower, and mass of the spleen was significantly greater (102). Further studies also suggest that a reduced oxidative capacity and lower capacity for fatty acid utilization in muscle tissue underlie insulin resistance in South Asians, but also indicate that this is not due to differential gene expression (103).

**Figure 8:**
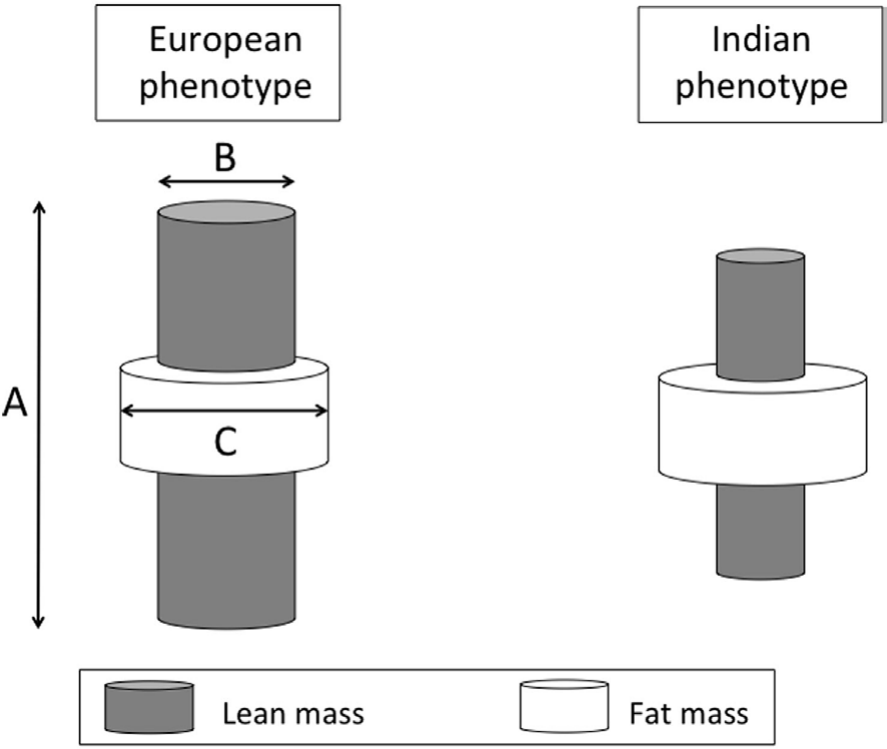
**Schematic diagram illustrating diabetes risk as a function of three dimensions of body size**. The length (A) and cross-sectional area (B) of an internal cylinder of lean mass is considered a marker for metabolic capacity, whereas the volume of the external cylinder of fat mass (C) is considered a marker of metabolic load.

One consequence of this difference in physique is that for any given BMI value, Indians have a higher proportion of fat mass in their body weight relative to Europeans (93–95). We can, therefore, highlight several ways in which Europeans and Indians differ in their size, shape, and body composition, using a simple cylinder model where each of the three primary dimensions indexes one component of diabetes risk (Figure 8). The inner cylinder represents lean mass, and both the height and cross-sectional area of this cylinder (associated with skeletal dimensions) are proxies for metabolic capacity. The outer cylinder represents body fat (much of which is located in subcutaneous depots although some is located in deeper internal depots) which provides a proxy for metabolic load.

**Figure 9:**
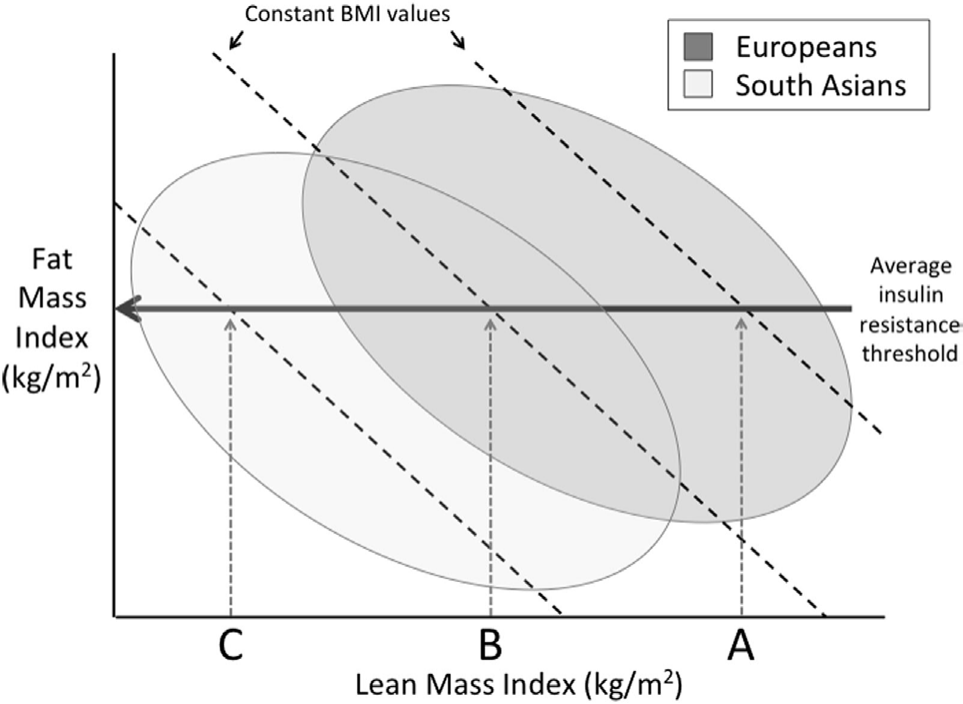
**Schematic diagram illustrating differences between Europeans and South Asians on a plot of fat mass index (fat mass divided by height squared) and lean mass index (lean mass divided by height squared), in which the sum of these two traits is equal to body mass index (BMI)**. Above a certain threshold of fat mass index, insulin resistance develops. However, due to their lower lean mass index, Indians develop insulin resistance at lower levels of BMI compared to Europeans.

Much attention has been given to the relatively greater adiposity of Indians, as it elevates insulin resistance and, hence, diabetic risk (13). Moreover, due to the higher fat–lean ratio, indicating a greater load–capacity ratio, increases in BMI in Indian populations elevate diabetes risk to a greater extent than they do in European populations, raising the susceptibility to insulin resistance (Figure 9). In a longitudinal study of adults in New Delhi, for example, relatively modest gains over time in BMI increased diabetes risk in the absence of overt obesity (104). Because body fat typically increases with age through adult life, this also offers an explanation for why Indians typically develop diabetes at younger ages than Europeans – Indians are predicted to reach a harmful threshold of metabolic load earlier (104). Beyond this greater ratio of fat mass to lean mass, Indians also have greater metabolic sensitivity to adipose tissue than other ethnic groups. For example, a given mass of fat generates greater insulin resistance in Indian compared to European children (105).

Relative to Europeans, therefore, contemporary Indians are both “fat” and “thin,” and this phenotype has been observed at birth and during early infancy (106–108), indicating that it emerges within the niche of maternal pregnancy physiology. Mean birth weight among Britons of Indian ancestry is ~3.1 kg compared with 3.4 kg among white British newborns (109), and birth weight in India itself averages ~2.9 kg (110). The low birth weight Indian baby shows relative preservation of the head and subcutaneous body fat at the expense of muscle tissue and viscera, indicating particularly low non-brain organ masses at birth (106). Thus, during fetal life, Indian neonates develop a reduced metabolic capacity compared to Europeans and a relatively higher metabolic load (108, 111).

These traits are indicative of reduced maternal nutritional investment, and additional data support this hypothesis. In humans in general, short maternal stature is associated with lower birth weight (112), while a flatter maternal pelvis also constrains fetal growth (113). Specifically in Indians, shorter maternal stature and lower BMI are associated with lower birth weight (114–116), while the dimensions of the maternal pelvis in Indians are smaller than in European populations (117, 118). Maternal lean mass may be the primary physical determinant of offspring birth weight (119). In several ways, therefore, the small size of Indian mothers constrains nutritional investment in the fetus.

Fears of cephalo-pelvic disproportion, which could obstruct labor, promote the custom of pregnant women “eating down” (deliberately reducing food intake) toward the last trimester of pregnancy (120). There is minimal documentation of the net impact of this practice, yet it is commonly reported in anthropological and other qualitative studies in South Asia (120–122). Nevertheless, evidence shows that maternal nutritional supplementation during pregnancy need not in fact lead to cephalo-pelvic disproportion (123). The magnitude of such supplementation on birth weight is typically <100 g except where mothers are severely undernourished (124–128). On the other hand, a recent supplementation study in India that began pre-pregnancy had no effect on birth weight in underweight mothers, but increased birth weight by ~90 g in mothers with baseline BMI > 18.5 kg/m^2^ (129). Thus, the effect of increasing maternal dietary energy supply appears to depend on whether it occurs before or during pregnancy, but a common finding is that effects on birth weight are modest.

Beyond maternal size, various maternal micronutrient deficiencies have been shown to limit fetal weight gain (130). Recent work in a rural cohort from Pune has highlighted low intake vitamin B12 and functional folate deficiency as maternal dietary determinants of low birth weight, mediated through their impact on maternal homocysteine levels (131). The longer-term influence of these maternal micronutrient deficiencies on diabetes risk is shown by an association between low maternal B12 during pregnancy and insulin resistance in the offspring at 6 years, exacerbated by high folate levels (130). Vitamin B12 acts as a marker of non-vegetarian dietary intake; hence, these data provide support for a detrimental impact of maternal vegetarian diets on the development of metabolic capacity in the offspring.

Prenatal and early infant growth appears to be the critical period for determining lean mass. If energy supply increases from early childhood onward, there is little opportunity for it to be invested in organ structure and metabolic capacity, hence, it must be diverted to other life history functions (growth, reproduction, immune function) (132). These other functions are strongly mediated by elevated body fat, which promotes faster maturation and immune function, but at a cost of elevated metabolic load. In other words, improved energy supply from childhood onward cannot resolve the “thin” component, and primarily increases the “fat” component of the thin–fat phenotype.

Thus, the high diabetic risk of contemporary South Asians may be explained in terms of a reduced metabolic capacity that renders them particularly susceptible to the high metabolic load associated with the lifestyles that track urbanization, or migration to higher-income settings (133). The question of when and why this lower stature and lean mass originated among South Asians may be approached by examining long-term trends in stature and physique in South Asia by means of archeological and historical evidence, and by considering the environmental factors that may have driven evolutionary change, whether through genetic adaptation or trans-generational plasticity. Although soft tissue itself is very rarely preserved in the archeological record, the relationship between stature, metabolic capacity, and diabetic risk described above, as well as more scarce indicators of physique (muscularity and body mass), enable us to attempt to reconstruct long-term trends in metabolic capacity in South Asian populations.

## Archeological Evidence for Trends in Stature and Physique

India’s archeological skeletal record embraces several distinct subsistence patterns, including Mesolithic forager, Neolithic–Chalcolithic agro-pastoralist, Iron Age Megalithic, and early Indus Valley urban populations (134). From these data, broad long-term trends in skeletal morphology can be discerned, though the populations are also distributed unequally across the subcontinent and their precise ancestral relationships are unknown.

Foraging during the Mesolithic period was associated with robust dentition, indicating consumption of a coarse, high-fiber diet (134). From around 8000 BP, foraging was gradually replaced by food production, resulting in crop cultivation (earliest evidence from Mehrgarh, contemporary Pakistan, 8500 BP) and animal domestication (earliest evidence from Bagor, south Rajasthan, 6600 BP). Food production had become the primary mode of subsistence by 4000 BP in the fertile north, but took longer to consolidate in the peninsular south due to less favorable climate and topography (134). The shift to agriculture promoted sedentary living, initially in small-scale villages that in some regions, such as the Indus valley, developed into larger urban settlements (135). Sedentary living decreases inter-birth interval and, therefore, favors population growth (136).

Over the period when agriculture emerged, developmental lesions in dental enamel (hypoplasias) indicate growth interruptions in both foragers and farmers from the northern plains, but the tall stature attained by the foragers suggests that compensatory growth was possible and there are negligible signs of overt nutritional deficiencies or heavy infectious disease loads (137, 138). These characteristics indicate that the foragers were healthy and well-adapted to their ecological niche, in contrast to subsequent agriculturalists (139).

Early agricultural populations were characterized by more gracile skeletal structure, implying reduced musculature (134). These changes can be attributed both to push factors (decreased mechanical stress from a less coarse diet, decreased habitual mobility) and to pull factors (increased prevalence of nutritional deficiencies and infectious diseases) constraining growth (140, 141). Compared to the average stature of male Mesolithic foragers of 181.5 cm, the Chalcolithic males averaged only 172.5 cm (137), an extreme difference in body morphology between the two subsistence regimes.

Paleo-ethnobotanical data indicate similar contrasts between the nutritionally rich and varied diet of Mesolithic foragers, and the carbohydrate-rich but nutrient-poor diet of early farming communities (142). Crops providing the highest energy returns have low nutrient densities (143), and the downward shift in stature and skeletal proportions mimics a similar trend in early farmers worldwide (144–148), although the large magnitude of decline and its persistence across many millennia in the Indian subcontinent appear atypical (144). Historical data from Europe indicate a close link between infectious disease burden in early life and reductions in adult height (149). Early Indian farming populations show evidence of periodic famines, food shortages, and high infection rates, along with ecological stress from over-use of the land, all well-recognized features of early agricultural life (134). These factors may have collectively contributed to their shorter stature and skeletal gracility.

The Neolithic and Chalcolithic village agriculture that flourished during the period 6000–4000 BP is not dissimilar to that practiced by Indian rural society up to the late twentieth century. Gradual technological change and the emergence of occupational castes consolidated the settled agricultural niche and favored further urbanization in the north around 2600 BP (134). The archeological evidence indicates a life expectancy at birth of <20 years in early agricultural populations, due in large part to high levels of infant mortality. Such mortality rates are typically countered by high fertility rates, and this is consistent with a substantial increase in population size following the introduction of iron technology. Even until recent times, a common traditional blessing for an Indian bride was the hope for eight offspring.

Beyond its association with falling adult stature, the combination of population growth and increased infectious disease burden implies changes in fertility rates and, hence, size at birth. In turn, shifts in life history and reproductive strategy – from fewer, larger to more, smaller offspring – imply adaptations in insulin metabolism, as this hormone orchestrates fundamental associations between growth, maturation, reproduction, and social behavior (132, 133, 150, 151). Thus, the emergence of agriculture appears to have played a key role in the long-term transition to smaller body size, but other ecological factors are also likely to have contributed.

## The Emergence of Vegetarianism: Decline in Protein Consumption

Following the transition to agriculture, the emergence of vegetarianism is likely to have exerted an additional dietary stress. From early Hindu religious texts, it is clear that animals were initially sacrificed and meat eaten. By 600 BCE, however, major changes had occurred in religious thought. It has been suggested that, by this time, war, drought, and famine were becoming increasingly common, giving the impression that the old Vedic gods were failing (152). Population growth and the need for more land to feed people were also stressing the agricultural economy. The Zebu humped cow began to be considered too valuable for economic and agricultural survival for it to be sacrificed or consumed. These ideals began to enter the texts known as the Upanishads from around 800 BCE.

Around 600 BCE, an even more ascetic tradition known as Jainism emerged. Rejecting many elements of Hinduism, Jainism requires total non-violence, and prohibits the consumption of meat, eggs, and fish (152). Some contemporary Jains also avoid milk products and consume vegan diets. A similar drive toward vegetarianism followed the emergence of Buddhism in the fifth century BCE. Buddha specifically condemned all killing, war and aggression, and banned animal sacrifices and trade in animal products (152). Buddhism flourished during the 37-year rule of the emperor Asoka, starting in 265 BCE, by which time the Magadhan Empire founded in the sixth century BCE had expanded into a territory almost the size of modern India.

Through the influence of Hinduism, Jainism, and Buddhism, vegetarianism became widespread in India and has been widely practiced for over 2000 years. On the basis of recent empirical studies, its low-protein regime is likely to have impacted growth and development. This is especially relevant to growth patterns in early life as, in accordance with Hindu cultural traditions whereby males eat before females, the *de facto* level of vegetarianism is higher in females than males (though not through choice). Maternal protein intake in early pregnancy is positively associated with birth weight (153, 154), and a study of UK children born to vegan mothers reported reduced weight and stature, compared to national growth standards (155). Dietary protein intake in infancy (156–158) and childhood (159–161) are also positively associated with rates of growth in weight and stature, although whether either of energy or protein is a limiting factor for infant growth may depend on the dietary availability of the other (162).

Another constraint on growth may derive from a general inability to digest lactose. Milk is a particularly important driver of growth in humans, providing not only protein but also other growth-promoting factors. Tall statures in east African pastoralist populations have been attributed to high milk intakes (163), consistent with data from western populations (160, 164). Significantly, however, Indian populations are relatively lactose-intolerant, although much more so in the south, where the prevalence of primary lactose intolerance is ~70%, compared to the north, where the value is ~30% (165). Pastoralist castes, such as the Toda, who have adapted to drink milk, have ~10 cm taller stature than other castes in the same region (166), while in the Pune Maternal Nutrition Study, maternal milk consumption in pregnancy was positively associated with the offspring’s length at birth (167). Consumption of camel milk was associated with zero risk of diabetes in north-west Rajasthan (168), although milk consumption was not associated with diabetes risk in a heterogeneous national sample (169).

There is a related emerging literature on infection reducing absorption of protein when it is of vegetable origin. Given the high infection load found among Indian infants, this suggests that malabsorption of lower quality vegetable protein exacerbates their growth problems (170, 171).

Thus, the combination of low-protein vegetarian diets, micronutrient-deficient diets, and an inability to digest milk is likely to have constrained growth and body size in the Indian population in recent millennia. There is a notable north-south gradient in diabetes prevalence in India (12, 172), and the taller stature of northern populations may protect against diabetes, with greater lactose tolerance one potential underlying mechanism.

## Ecological Disruptions to the Food Supply

Beyond long-term subsistence trends, nutritional status is also affected by acute events and their long-term patterning. Indian agriculture is heavily dependent upon monsoon rainfall to water the crops, both as the monsoon clouds drift north in the early summer and as they return in the early autumn. The failure of the monsoon rains in El Niño years decimates crop harvests across wide geographical regions, and these effects may then interact with socio-economic phenomena, inducing substantial increases in the price of food. Particularly severe famines were documented in 1769–1770, 1876–1878, 1896–1897, and 1899–1902, resulting in millions of deaths on each occasion (173, 174). In 1943, the Bengal famine is estimated to have killed between 1.5 and 4 million (175).

While the proximate cause of Indian famine is monsoon failure, rates of mortality increased sharply in the Victorian era through failure of the British to distribute famine relief. Prior to the arrival of the British, the Mogul empire was relatively efficient at redistributing food during periodic monsoon droughts. Famine appears to have become much more frequent during the British era, and although climatic trends might have contributed to this, there is strong evidence that the economic influence of the British exacerbated the calamitous toll in morbidity and mortality (174).

These famines are likely to have acted harshly on body size, potentially through different biological processes. On the one hand, high levels of mortality are predicted to have selected for genotypes conferring smaller size, which would require fewer resources to grow and survive. On the other hand, downward secular trends through trans-generational phenotypic plasticity are also likely to have contributed. The relative contributions of these two processes remain unknown; however, upward secular trends in stature and maturation rate in the Indian population in recent decades give an indication that trans-generational plasticity is relevant (176, 177).

Famine might conceivably have selected in favor not only of smaller size but also of metabolic adaptations and an increase in the ratio of fat mass to lean mass, i.e., the thin–fat phenotype. This hypothetical scenario fits closely with empirical evidence reviewed above. Recent studies have shown associations between a variety of specific alleles and the risk of diabetes or obesity in Indian populations (23, 24, 178), with the pattern of association not always directly equivalent to that in Europeans.

## Historical Evidence on Stature Trends from Indentured Laborers

While data on stature trends between the adoption of agriculture and the early modern period are sparse, the first historical measurements of stature in the nineteenth century show clearly that South Asians were short compared to the average in most other global regions (179). In the late nineteenth and early twentieth centuries, male stature in India showed a north-south gradient, with populations in the peninsula south averaging ~161 cm and those in the north averaging ~167 cm (166). This gradient may relate both to moderate climatic adaptation, and also to different diets, but even the northern populations are ~15 cm shorter than the Mesolithic foragers discussed above, as well as more gracile (134, 137).

**Figure 10:**
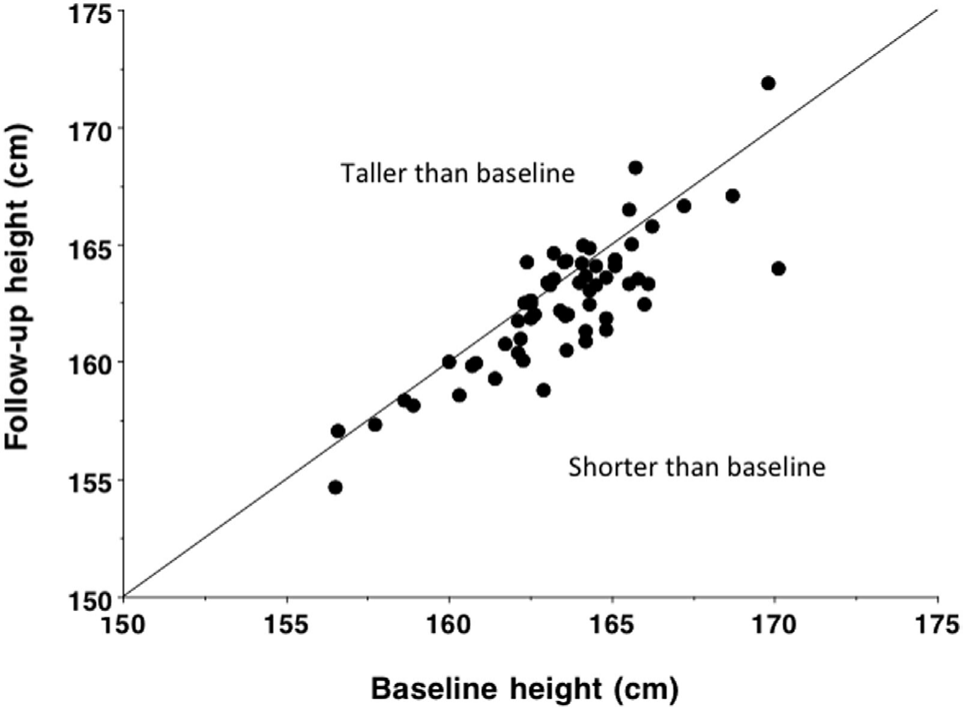
**Baseline and follow-up height in 60 populations of male Indians, measured over an average time interval of 48 years between 1881 and 1962 and equivalent to an average decline of 9.1 mm (*p* < 0.0001)**. Based on data from Ganguly (182). Reproduced with permission from Ref. (133).

Moreover, unlike in other global regions, continuing declines in stature in India are evident over the last two centuries. Data on stature were routinely collected from those who left India under the indentured labor scheme in the early nineteenth century, to address the shortage of labor on colonial plantations following the abolition of slavery. For approximately a century, almost 1.2 million Indians signed short-term contracts and were transported to the Caribbean, Fiji, Mauritius, and east and southern Africa. Records obtained at recruitment show either no upward increase in stature over this period, or a slight secular decline (180, 181). Further smaller-scale longitudinal studies of individual populations suggest an average decline in male stature between 1881 and 1962 equivalent to −1.8 cm per century (Figure 10) (182). These data contrast markedly with the substantial upward trend in stature that characterized industrialized populations during the twentieth century (183).

## Long-Term Trends in Birth Weight

The low birth weight of contemporary Indian populations is, therefore, likely to be a product of the long-term downward trend in height. Both within and between populations, birth weight scales with maternal stature (112, 184). This scaling association can be used to predict birth weight in past populations from the skeletal evidence. Such an approach predicts that birth weight in India would have been ~3440 g in the early forager populations of 8000 years BP, ~3200 g in early farming populations of 4000 years BP, compared to ~2800 g today. These simulations suggest a 20% reduction in birth weight over the last 10,000 years, at an average rate of 6.4 g per century.

Studies of birth weight among those born with similar or contrasting parental ethnicity may shed more light on the mechanisms that underlie this decline (185). In an analysis of data from the UK, average birth weight of offspring with two European parents was 344 g greater than that of offspring with two Indian parents. Compared to offspring of European mothers, the offspring of Indian mothers had lower birth weight, whether the father was European (Δ = 152 g) or Indian (Δ = 254 g). This clearly indicates a maternal ethnic influence, but it is difficult to ascribe it with confidence to a genetic or environmental basis. However, beyond this, it was also possible to determine paternal influences on birth weight.

After adjustment for various confounding factors, average birth weight of offspring with European father and Indian mother was 249 g greater than that of offspring with two Indian parents. Conversely, average birth weight of offspring with Indian father and European mother was 117 g less than that of offspring with two European parents. These paternal associations suggest that any nutritional constraint of the Indian mother is not “fixed,” and can be partially overcome if a higher level of nutritional investment is “demanded” by paternal genotype. On the other hand, Indian paternal genes appear to have adapted to demand lower fetal nutritional investment. The implication is that long-term nutritional experience over generations may drive paternal co-adaptation, through either genetic or epigenetic mechanisms.

## Secular Trends in Recent Decades

Upward trends in body size occurred in many high-income countries through the nineteenth and twentieth centuries, the best-known example being Holland where male height increased by ~22 cm over 135 years, or 1.63 cm per decade (186). The most recent data indicate the beginning of a similar trend in the Indian population, especially in urban populations since the 1960s (176), but the rate of change has been relatively modest relative to European populations.

Over 25 years, height increased by ~0.50 cm/decade in Indian men, barely one-third of the rate in Holland (187). In Indian women, the trend was ~0.16 cm/decade, less than an eighth of that imputed for Dutch women. The trend also varied across states, ranging from negative rates of −0.37 cm/decade in Delhi, −0.17 cm/decade in Bihar, and −0.22 cm/decade in Nagaland to positive rates of 0.66 cm/decade in Tamil Nadu and 1.16 cm/decade in Kerala (176). These trends are unlikely to reflect variable effects of aging and changes in posture, as the samples were ≤49 years of age in women and ≤54 years in men. Rather, negative trends in females might be linked with a shift toward earlier puberty (188) in association with slightly shorter final stature, as reported in Indian migrants to Sweden (189), though this issue merits further investigation. These individuals were born between the 1960s and 1980s, and the secular trend may since have accelerated, but the long-term contrast with European populations is still striking. Secular increases in birth weight are also very slow, equivalent to a change of 1 SD score across 15.5 decades (190).

**Figure 11:**
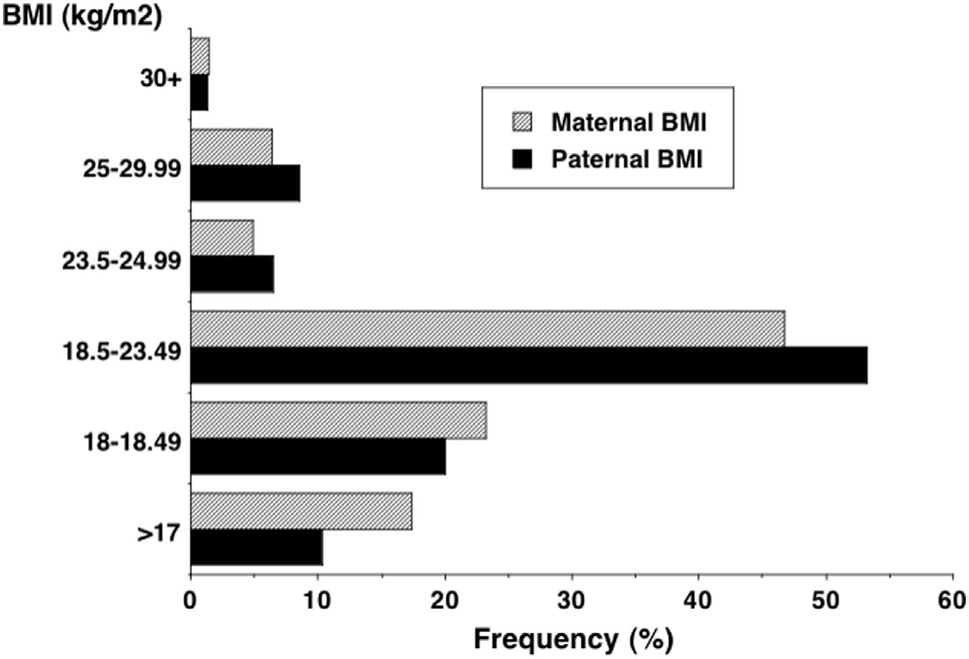
**Distribution of adult BMI in the 2005–2006 Indian National Family Health Survey, showing that 10% of both fathers and mothers exceeded 23**.**5 kg/m^2^, the cut-off for overweight in the Indian population (192)**.

Aside from such limited changes in stature, data from recent family health studies indicate more substantial increases in average BMI (9, 191). Figure 11, for example, shows that over 10% of both fathers and mothers in the 2005–2006 Indian National Family Health Survey had BMI in excess of 23.5 kg/m^2^, the cut-off for overweight in the Indian population, and that almost 50% of fathers and mothers had BMI between 18.5 and 23.5 kg/m^2^ (192). Using population-specific BMI cut-offs, the rate of obesity in Delhi adults was already 21.3% in males and 33.4% in females in 1994, and by 2011 this had risen to ~50%, a pattern repeated in other cities, such as Chennai (193–195).

**Figure 12:**
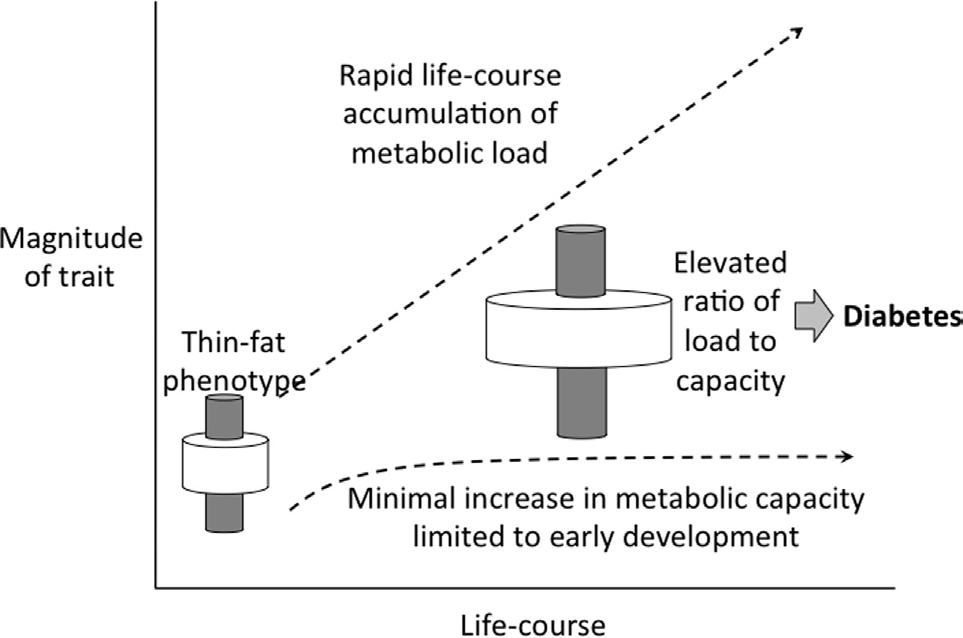
**Schematic diagram illustrating how the elevation in metabolic load, generated by rapid increases in BMI and adoption of unhealthy lifestyle, is not matched by a recovery of metabolic capacity, dependent on long-term trends in birth weight**.

The disparity between these trends in birth weight or stature and BMI mean that metabolic load is rapidly increasing relative to metabolic capacity (Figure 12). This provides an explanation for why the Indian diabetes epidemic emerged only recently: it is only when metabolic load is high that the susceptibility of low capacity is activated.

## Conclusion

Our review of long-term trends in stature in the Indian population, interpreted through the lens of the capacity–load model described earlier, offer an explanation for the elevated risk of diabetes in contemporary Indians. Due to their relatively higher ratio of fat to lean mass for any given level of BMI, Indians accumulate a higher metabolic load when their BMI increases. In addition, they have a reduced capacity to tolerate this load, given their low average birth weight and their reduced ability to clear glucose, deriving from the lower lean mass. The younger average age of diabetes onset in Indians than in Europeans also indicates reduced metabolic resilience.

Our evolutionary perspective indicates that these ethnic metabolic differences arise in large part from contrasting long-term nutritional histories. The drastic reduction in stature over the last 10,000 years in India is greater than that observed in most other global regions (144), and indicates nutritional stress operating across many generations.

These characteristics now contribute to the high vulnerability of contemporary Indians, both in urban populations within India and in emigrant populations overseas, to the obesogenic factors now prevalent in urban populations worldwide. Increased energy availability in contemporary Indians is resulting in rapid secular increases in BMI and abdominal obesity (8, 193–195). According to our approach, the long-term decrease in metabolic capacity has been so substantial that even moderate increases in load induce substantial metabolic penalties. This is consistent with data showing that even modest BMI increases are associated with an increased risk of diabetes, the metabolic syndrome, and cardiovascular disease (104).

The challenge is how to escape this vicious cycle. While in theory, increased nutritional investment by the mother during pregnancy may protect against diabetes in the offspring, in practice greater maternal nutritional intakes during pregnancy may overload maternal glycemic control, and promote gestational diabetes. Furthermore, short mothers are at much greater risk of gestational diabetes than taller mothers (196, 197). This highlights how reduced metabolic capacity in one generation increases diabetic risk in the next. In turn, maternal obesity and diabetes are important risk factors for macrosomic offspring (198).

The implications of our perspective are that public health programs must be realistic about what beneficial phenotypic changes can be achieved in the short term. Decreases in metabolic capacity occurred over millennia, probably through a combination of changes in gene frequencies, selection of new mutations, and non-structural epigenetic effects and other physiological changes contributing to trans-generational plasticity. While increases in birth weight, adult stature, maternal pelvic dimensions, and lean mass would all enhance metabolic capacity, such changes are only likely occur to incrementally across multiple generations (132, 199, 200), and in Indian immigrants in the UK, negligible increases in birth weight have occurred across generations (201, 202). Although secular increases in birth weight might occur, substantial changes in maternal BMI are likely to provoke gestational diabetes rather than beneficial birth weight increments.

In the short term, therefore, public health policies to constrain the exacerbation of metabolic load from early childhood onward are paramount. Efforts should prioritize the prevention of excess weight gain and abdominal obesity, the prevention of sedentary behavior, and a shift toward diets with low glycemic index and lower fat content. A particular challenge concerns how to achieve such reductions in metabolic load, while also promoting increases in metabolic capacity in the next generation. For example, the persisting problem of child malnutrition in India and other South Asian countries is increasingly addressed through the provision of ready-to-use therapeutic foods (RUTF) (203), which are designed for rapid short-term weight gain. While these undoubtedly improve immediate survival, their long-term implications for adult chronic disease risk remain unknown.

## Author Contributions

JW, BP, and CY conceived the original idea, and developed it in collaboration with EP and SW. JW wrote the first draft of the article. All authors contributed to revisions.

## Conflict of Interest Statement

The authors declare that the research was conducted in the absence of any commercial or financial relationships that could be construed as a potential conflict of interest.
